# Pet attachment and prosocial attitude toward humans: the mediating role of empathy to animals

**DOI:** 10.3389/fpsyg.2024.1391606

**Published:** 2024-06-12

**Authors:** Jhon Marc V. Faner, Ethel Ann R. Dalangin, Lei Ann Trishia C. De Leon, Levi D. Francisco, Yessamin O. Sahagun, Evelyn F. Acoba

**Affiliations:** Department of Psychology, College of Arts and Social Sciences, Central Luzon State University, Science City of Munoz, Nueva Ecija, Philippines

**Keywords:** human–animal interaction, attachment, animal empathy, prosocial attitude, mediation analysis

## Abstract

Attachment relationships are widely recognized as influential in increasing prosocial tendencies, with existing literature indicating that human attachment can increase empathetic processes, thereby potentially facilitating prosocial behavior. Given that pets frequently fulfill the criteria for attachment figures, this study investigates whether the observed associations among human attachment, empathy, and prosocial attitudes extend to human-animal interactions (HAI). This study examines the relationship between pet attachment, animal empathy, and prosocial attitudes toward humans. The study hypothesizes that animal empathy mediates the association between pet attachment and prosocial attitudes. A cross-sectional survey was administered to 343 Filipino participants, predominantly consisting of single female young adults with college education backgrounds. Participants completed a battery of assessments including the Contemporary Companion Animal Bonding Scale (CCABS), the Animal Empathy Scale (AES), and the Prosocialness Scale for Adults (PSA). Aligned with our hypothesis, our study reveals that animal empathy plays a significant mediating role in the relationship between pet attachment and attitudes toward humans. We found that stronger pet attachment correlates positively with heightened animal empathy, subsequently leading to elevated levels of prosocial attitudes. Our findings prompt discussions on implications for understanding human-animal relationships and suggest avenues for future research exploration.

## Introduction

Attachment theory posits that individuals are born with behavioral systems primarily designed to promote survival (Bretherton, 1992; Gillath et al., 2005; Jones, 2015; Shaver et al., 2016; Stern and Cassidy, 2018). Two key systems within this framework are the attachment behavioral system and the caregiving behavioral system (CBS). The attachment behavioral system is activated during stressful situations to regulate distress by seeking responsive and sensitive care from attachment figures. This system drives individuals to seek proximity to attachment figures, and achieving this proximity elicits feelings of relief and security (Bretherton, 1992; Gillath et al., 2005; Shaver et al., 2016). When coupled with attentive caregiving, this leads to the development of a positive internal working model (IWM) of the self as a valued and competent individual (Gillath et al., 2005; Stern and Cassidy, 2018). Furthermore, perceiving an attachment figure as attentive helps mold an IWM of others as worthy of care (Hawkins et al., 2017). This mental representation influences individuals to be empathetic toward their source of comfort during times of stress, fostering and cultivating empathetic responses within attachment relationships (Bretherton, 1992; Gillath et al., 2005; Panfile and Laible, 2012; Costa Martins et al., 2022; Xu et al., 2022).

Complementing the attachment behavioral system, the caregiving behavioral system (CBS) aims to provide responsive and sensitive care to distressed individuals (Bretherton, 1992; Jones, 2015; Shaver et al., 2016). The CBS organizes caring behaviors intended to alleviate stress by serving as a safe haven and secure base for those exhibiting distress cues (George and Solomon, 2008; Shaver et al., 2016). This system is inherently altruistic, designed to support and nurture others in need (Gillath et al., 2005). When the caregiving behavioral system (CBS) is activated, it enables individuals to recall a repertoire of caring behaviors they have previously experienced (George and Solomon, 2008). This activation may explain why empathetic individuals are more predisposed to engage in prosocial behaviors.

Together, these systems form a theoretical framework for understanding the associations among attachment, empathy, and prosocial tendencies. The attachment behavioral system focuses on seeking care and forming secure bonds, while the CBS emphasizes providing care and ensuring the well-being of others. Both systems are crucial for developing healthy, supportive attachments and fostering empathetic and altruistic behaviors.

Numerous studies demonstrate a positive association between attachment and prosocial tendencies in both human-human and human-animal interactions (Thompson and Gullone, 2008; Langston, 2014; Hawkins and Williams, 2017; Malonda et al., 2019; Costa Martins et al., 2022; Berendzen, 2023; Deneault et al., 2023). Drawing from Bowlby’s attachment theory, which underscores the importance of emotional bonds, particularly between infants and caregivers, this paper delves into the foundational role of attachment in influencing prosocial tendencies. According to research, secure attachment, nurtured through responsive caregiving (Mikulincer and Shaver, 2005), not only influences individuals’ self-perception but also prompts caregiving behaviors (Gillath et al., 2005). Moreover, studies by Thompson and Gullone (2008), Schoeps et al. (2020), and Pan et al. (2022) consistently highlight empathy as a key mediator in the relationship between attachment and prosocial inclinations. These studies, conducted across various populations including Chinese, American, and Spanish adolescents, reveal a pattern wherein stronger attachments correlate with higher levels of empathy, subsequently leading to increased engagement in prosocial acts. With the growing recognition of pets as attachment figures (Kurdek, 2009; Zilcha-Mano et al., 2012; Rockett and Carr, 2014; Meehan et al., 2017) and their positive correlation with prosocial behaviors (Thompson and Gullone, 2008; Langston, 2014; Hawkins and Williams, 2017; Berendzen, 2023), coupled with an understanding of empathy’s pivotal role in linking attachment and prosocial tendencies observed in human-to-human interactions, this study aims to investigate whether empathy similarly acts as a mediator in the context of human-animal interactions.

### Pet attachment and prosocial attitude

Attachment, defined as the emotional connection between two individuals, was initially conceptualized as the relationship between a child and a mother (Zilcha-Mano et al., 2011; Wanser et al., 2019). However, some studies extend this concept to include the emotional bond between humans and pets. Attachment figures are not limited to humans and may also include pets (Mikulincer and Shaver, 2005; Kurdek, 2009; Zilcha-Mano et al., 2012; Rockett and Carr, 2014; Meehan et al., 2017). Rockett and Carr (2014) argue that pets can serve as both a secure base and a safe haven, offering reliable comfort and being sought out during times of distress. They assert that pets fulfill the need for proximity maintenance, with their physical presence providing a sense of safety, while their absence may trigger separation distress. Kurdek (2009) corroborates this by finding that individuals often seek solace in their dogs during emotional turmoil. Similarly, (Zilcha-Mano et al., 2012) confirmed that pets have the capacity to provide a sense of security and a dependable foundation.

Meanwhile, prosociality is mostly referred to as a desire for voluntary behavior intended to benefit others. Its aim is to help other people regardless of the benefits to the actor (Tur-Porcar et al., 2018; Costa Martins et al., 2022). Prosociality is argued to consist of actions such as helping, sharing, and taking care of individuals (Costa Martins et al., 2022). Prosociality is also referred to as the behavioral manifestation of empathy (Shaver et al., 2016). With the dawn of the COVID-19 pandemic, prosociality in the general population is identified to have been affected as well. During the onset, the negative impact of COVID-19 on prosociality was observed (van de Groep et al., 2020; Jin et al., 2021; Alvis et al., 2022). However, in the middle of the COVID-19 pandemic, the opposite was documented. It was reported that prosocial tendencies amid the COVID-19 pandemic served as a way of coping (Cho et al., 2021; Kislyakov and Shmeleva, 2021; Thu et al., 2021; Tse et al., 2021; Marinthe et al., 2022). However, the mechanism through which prosociality flourished during the pandemic remained unclear and may require additional investigation; thus, prompting this study.

Attachment research consistently provides robust evidence supporting a positive correlation between attachment and prosocial tendencies. Recent systematic reviews, such as that conducted by Costa Martins et al. (2022), consistently affirm this strong association, which closely aligns with Bowlby’s attachment theory. Costa Martins et al. (2022) suggest that secure attachment in childhood, characterized by a strong emotional bond with caregivers, enhances a child’s inclination toward concern for others, resulting in heightened levels of prosocial behavior. This assertion is also reported by Gross et al. (2017), who emphasize the diverse array of prosocial behaviors cultivated through secure attachment. Furthermore, Malonda et al.’s (2019) study further substantiates this connection, indicating that robust human attachment may serve as a predictor for positive prosocial outcomes. Additionally, Shaver et al. (2016) underscores the pivotal role of mindful, caring, and supportive parental figures in shaping children’s perceptions of others as competent, dependable, and benevolent, thereby reinforcing secure attachment patterns. Deneault et al.’s (2023) meta-analysis further support these findings, revealing a significant positive correlation between early childhood child–parent attachment and subsequent prosocial behavior. Collectively, these studies underscore the critical role of secure attachment relationships in nurturing altruistic tendencies and fostering healthy social interactions in children.

In the context of human-animal interactions, studies show a positive correlation between prosociality and pet attachment. Christian et al. (2020) conducted a longitudinal study revealing that children who own a dog exhibit enhanced prosocial tendencies over time. This finding aligns with Hawkins et al. (2017), who demonstrated that a strong attachment to pets significantly fosters prosocial behavior. Li (2022) further substantiated these results, reporting a positive association between pet attachment and prosocial behavior in children. Additionally, Zhou et al. (2010) identified that attachment to pets is positively correlated with improved self-concept, prosocial orientation, and daily living abilities in children. Collectively, these studies underscore the significant role of pet attachment in promoting prosocial behaviors in children.

Furthermore, certain studies offer insights into the mechanism of the positive correlation between pet attachment and pro-social behaviors. For instance, Wice et al. (2020) demonstrated the potential mediating role of empathy. Their research showed that engaging with pets was linked to increased dispositional empathy, which in turn correlated with enhanced pro-social behavior among children. Notably, this connection between compassionate relationships, empathy, and pro-social conduct was evident specifically when the animal was regarded as a companion rather than merely a recipient of basic caretaking (Wice et al., 2020). Thus, merely acquiring a pet does not automatically foster pro-sociality and empathy; instead, it is the depth of emotional connection with the pet that may nurture empathetic caregiving (Wice et al., 2020). Considering the documented associations between pet attachment and pro-social behaviors, this study posits that empathy toward animals may act as a mediator in the association between pet attachment and prosocial tendencies toward humans.

### Empathy and its potential mediating role

Empathy is broadly defined as a multidimensional construct encompassing the capacity to comprehend and share the emotional experiences of others (Decety et al., 2016; Stern and Cassidy, 2018; Sanctis and Mesurado, 2022). Affective empathy involves sharing the feelings or emotions of others, whereas cognitive empathy involves understanding and recognizing the emotions of others (Pérez-Fuentes et al., 2020; Thompson et al., 2022). Empathy and prosociality are related. According to Shaver et al. (2016), empathy is the emotional dimension felt by the individual regarding others’ welfare. On the other hand, prosociality is the active, behavioral manifestation of the said emotion that causes actions intended to benefit others.

Empathy is viewed as a product of attachment relationships (Xu et al., 2022). During stressful situations, attachment behavioral systems activate in individuals to regulate distress. This is characterized by seeking proximity to a potential attachment figure. In the attachment relationship, pets as attachment figures serve as representatives of entities other than the self (Panfile and Laible, 2012; Stern and Cassidy, 2018). As such, attentive caregiving provided of pets molds the internal working model of others (IWM) as worthy of care. The tendency of individuals to value their pet’s welfare may stem from acknowledgment of their pet as a source of comfort necessary to manage stress (Gillath et al., 2005; Shaver et al., 2016). Indeed, pets as attachment figures provide individuals with their need for a safe haven and secure base during periods of adversity. Hence, as individuals perceive their pet as worthy of care, this influences them to express empathetic concern for their source of comfort during stressful situations. Moreover, attachment theory suggests that the secure base function of attachment figures may motivate individuals to express empathy. By functioning as a secure base, attachment figures enable individuals to explore the emotions and perspectives of others without fear. Therefore, empathy is likely to develop as individuals are predisposed to engage in affective and cognitive empathetic processes (Sanctis and Mesurado, 2022).

Furthermore, empathy is acknowledged as a precursor to prosocial behavior. It is recognized as the primary motivator for altruistic actions (Lockwood et al., 2014; Stern and Cassidy, 2018). Empathy prompts individuals to extend comfort and assistance to those experiencing distress (Gillath et al., 2005), potentially modulating the operation of the caregiving behavioral system (CBS). The presence of empathy may heighten the responsiveness of the CBS, prompting individuals to offer sensitive and compassionate care to those in need (Shaver et al., 2016; Stern and Cassidy, 2018). Specifically, empathy enables individuals to readily discern distress in others and their requirement for support, thereby augmenting the activation of the CBS among empathetic individuals (Gillath et al., 2005; Decety et al., 2016). This activation enables individuals to draw upon a repertoire of nurturing behaviors learned through their own experiences (Shaver et al., 2010).

Empathy may not solely be expressed to individuals. The expression of empathy to animals was also reported (Vanutelli and Balconi, 2015). Stoeckel et al. (2014) suggest that empathy is not simply a response limited to humans alone but can also be extended to familiar animals. Moreover, empathy to animals was also found to be influential in an individual’s general empathetic processes (Vanutelli and Balconi, 2015). This is supported by the recent study of Gómez-Leal et al. (2021), which yielded a positive correlation between animal empathy and human empathy. The results complement the general assumption in the literature that animal empathy influences empathetic processes directed toward humans. Moreover, in recent studies, empathy is seen as having a mediating role in identifying the impact of attachment and prosociality (Schoeps et al., 2020; Costa Martins et al., 2022).

### The current study

**Figure 1:**
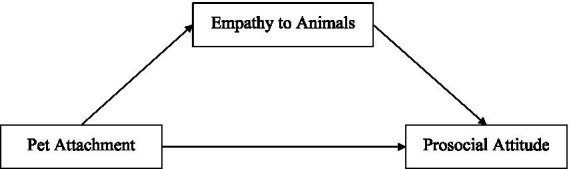
Schematic presentation of the proposed mediation model about the relationships among pet attachment, prosocial attitude, and empathy to animals.

Numerous studies on human attachment and prosociality provide evidence supporting the prevailing concept in attachment literature that it is linked to prosocial tendencies (Costa Martins et al., 2022). Because pets satisfy the prerequisites of attachment figures (Kurdek, 2009; Zilcha-Mano et al., 2012; Rockett and Carr, 2014; Meehan et al., 2017) this study posits that attachment to pets may also influence prosociality. Furthermore, the influence of pets on prosociality could be mediated by the empathy learned from human-pet interaction. Hence, this study aims: to determine the association between pet attachment and animal empathy; to determine the association between animal empathy and prosocial attitude; and to examine the mediating role of animal empathy on the association between pet attachment and prosocial attitude toward humans. The findings of the present study may provide valuable implications in further understanding the nature of HAI and its potential to promote prosocial attitude in individuals (Anderson, 2010; Sweijen et al., 2022) (Figure 1).

## Methodology

### Research design

The present study utilized a quantitative design, employing a correlational model to examine the association between pet attachment and prosocial attitudes. Additionally, the study investigated the potential mediating role of empathy toward animals in the relationship between these two psychological constructs.

### Ethical consideration

Approval from the Ethics Review Committee (ERC Code: 2022–323) was obtained before the study began, ensuring adherence to ethical guidelines. Participants received thorough briefings on the study’s details, including its nature and duration, to enable informed decision-making. They were informed of their rights to confidentiality, privacy, and the option to decline or withdraw from participation at any time. Stringent measures were taken to protect and securely store raw data, which were disposed of after processing to maintain confidentiality. Following data collection, participants received debriefing to address any questions or concerns. They were also provided with researchers’ contact information and access to mental health hotlines for additional support if needed. This comprehensive approach prioritized participants’ well-being and upheld ethical standards throughout the research.

### Participants

Participants in this study consisted of 343 Filipino adult pet owners currently residing in the Philippines with their pets. Their ages ranged from 18 to 33, with an average age of 20 years (SD = 1.613). The majority were single (75%) females (70%) attending public schools/colleges in Nueva Ecija, Philippines (91%), with a college education background (97%). Convenience sampling was employed to recruit participants, with the primary inclusion criterion being individuals currently living with their pets at the time of the study, aligning with the proximity requirement outlined in attachment theory (Gillath et al., 2005; Mikulincer and Shaver, 2005; Zilcha-Mano et al., 2011). The study focused on dog and cat owners, as advocated by Phillipou et al. (2021), who suggested that among pet types, dogs and cats share the closest similarities in terms of care requirements.

### Instruments

#### Contemporary companion animal bonding scale

The CCABS (Companion Animal Bonding Scale) was developed to gauge pet attachment (Poresky et al., 1987). It comprises 8 items rated on a 5-point Likert scale format, without any items scored in reverse. Scores on the CCABS are obtained by summing all responses, with higher scores indicating stronger pet attachment, and lower scores indicating weaker attachment. In a re-evaluation study by Triebenbacher (1999), three dimensions of the scale were identified: emotional bond, physical proximity, and caretaking, which align with how pet attachment will be approached in this study. Pet attachment, broadly defined, refers to the emotional bond between humans and their pets, with physical proximity and caretaking behavior serving as key considerations. The scale has demonstrated acceptable reliability, with internal consistency ranging from *α* = 0.82–0.77 in the original study and *α* = 0.80 in Triebenbacher’s re-evaluation. However, in the present study, the coefficient alpha yielded a lower value (*α* = 0.73) than reported in previous studies. Nevertheless, confirmatory factor analysis was also performed to examine how well the data fit the one factor model of the scale. Fit indexes indicate adequate fit (CFI = 0.73; TLI =0.62; RMSEA = 0.18).

#### Animal empathy scale

The Animal Empathy Scale (AES), developed by Paul in 2000, is designed to gauge empathy toward animals. It consists of 22 items rated on a 9-point Likert scale. The scale distinguishes between unempathetic and empathetic responses, with certain items being reverse-scored. Total scores on the scale are calculated by summing all responses, where higher scores indicate greater empathy toward animals, and lower scores indicate less empathy. Previous research has shown the scale to possess good internal consistency, with reported reliability coefficients of *α* = 0.78 (Paul, 2000) and *α* = 0.87 (Gómez-Leal et al., 2021). However, in our current study, the alpha coefficient was found to be lower but still acceptable (*α* = 0.66) compared to earlier findings. Despite this, the Animal Empathy Scale remains suitable for our research due to its alignment with our study objectives, sufficient item count, and known variability in reliability across different contexts. Additionally, past studies have validated the scale for measuring empathy toward animals.

#### Prosocialness scale for adults

The Prosocialness Scale for Adults (PSA), developed by Caprara et al. (2005), is designed to measure an individual’s prosocial attitude. It consists of 16 items rated on a 5-point Likert scale format, with scores determined by calculating the mean. Higher scores on the PSA indicate a stronger prosocial attitude, while lower scores indicate the opposite. Unlike most prosocial scales, the PSA specifically targets the adult population, focusing on actions such as sharing, helping, taking care of, and feeling empathetic toward others, as suggested by existing literature (Caprara et al., 2005; Costa Martins et al., 2022). The scale has demonstrated high reliability, with reported internal consistency coefficients of *α* = 0.95 by the authors. Similarly, the present study also reported high reliability of the scale (*α* = 0.91).

### Data gathering procedure

Prior to commencing data collection, the study underwent thorough review and approval by the Ethics Review Committee. The data gathering process involved the use of both traditional paper-and-pencil surveys as well as an online format administered through Google Forms. The survey itself was divided into three main sections. The initial section included an informed consent form, outlining pertinent details about the study and ensuring participants’ understanding and voluntary participation. The second section encompassed socio-demographic inquiries such as age, sex, year level, relationship status, number of pets, and the specific type of pet participants owned. This section also delved into whether participants currently shared their living space with their identified closest or most attached pet. Participants were requested to think of their closest pet as they answer the questionnaire. Lastly, the third section of the survey featured the administration of three instruments: the Contemporary Companion Animal Bonding Scale (CCABS), the Animal Empathy Scale (AES), and the Prosocialness Scale for Adults (PSA). The study used the original English versions of the instruments.

Before conducting data analysis, thorough data cleaning procedures were executed, with responses being excluded based on several criteria, including respondents not meeting the study’s specified criteria and missing data on the psychological scales. A notable observation was that most excluded responses came from the paper-and-pencil survey format, which exhibited a higher frequency of missing data compared to the online format. Toward the latter stages of the COVID-19 pandemic, as lockdown policies were relaxed and face-to-face classes resumed, a paper-and-pencil survey format was adopted to enable faster and more abundant responses. This survey was conducted within the rural province of Nueva Ecija.

### Data analysis

This study employed SPSS for data analysis, employing various statistical techniques to explore the dataset comprehensively. Initial analyses entailed verifying the assumptions of normality, linearity, and homoscedasticity. Raw data, rather than standardized values, were used for the analysis. Descriptive statistics were then calculated to provide a clear overview of the characteristics of the gathered sample population, including measures of central tendency and dispersion. Furthermore, to examine the mediating effect of empathy toward animals on the association between pet attachment and prosocial attitude, Hayes PROCESS Macro Model 4 was utilized (Hayes, 2012). This model allowed for a detailed examination of the indirect effects of pet attachment on prosocial attitude through empathy toward animals, while controlling for relevant covariates. By employing Hayes PROCESS Macro Model 4, the study aimed to elucidate the underlying mechanisms through which pet attachment influences prosocial behavior, shedding light on the role of empathy toward animals in increasing positive social attitudes.

## Results

The primary aim of the present study was to examine the impact of pet attachment on prosocial attitude. The findings of the study are detailed below. Table 1 shows the descriptive statistics and correlations of the study variables. Findings revealed linear associations between the study variables. Pet attachment displayed a linear association with both animal empathy (*r = 0.*22, *p* < 0.001) and prosocial attitude (*r = 0.*25, *p* < =0.001). Similarly, a linear association was also present between animal empathy and prosocial attitude (*r* = 0.24, *p* < 0.001).

**Table 1:** Descriptive statistics and correlations of study variables.

Variables	1	2	*M*	*SD*
1.Pet attachment			31.39	4.64
2.Animal empathy	0.22***		139.19	15.5
3.Prosocial attitude	0.25***	0.24***	4.02	0.57

***p < 0.001.

Table 2 presents the results of a simple linear regression analysis examining the relationship between pet attachment and prosocial attitude. The findings indicate that pet attachment accounted for 6% of the variance in prosocial attitude [*R*^2^ = 0.065, *F*(1, 341) = 23.553, *p* < 0.000]. This suggests that while pet attachment has a statistically significant effect on prosocial attitude (*β* = 0.031, *t* = 4.853, *p* < 0.001), its predictive capacity is relatively modest.

**Table 2:** Simple linear regression model for pet attachment and prosocial attitude.

Model	Unstandardized coefficients		Standardized coefficients	*t*	*p*
	*B*	Std. Error	Beta		
Pet attachment	0.031	0.006	0.254	4.853	0.000

Model *R*^2^ = 0.065, *F*(1, 341) = 23.553, *p* < 0.001.

**Figure 2:**
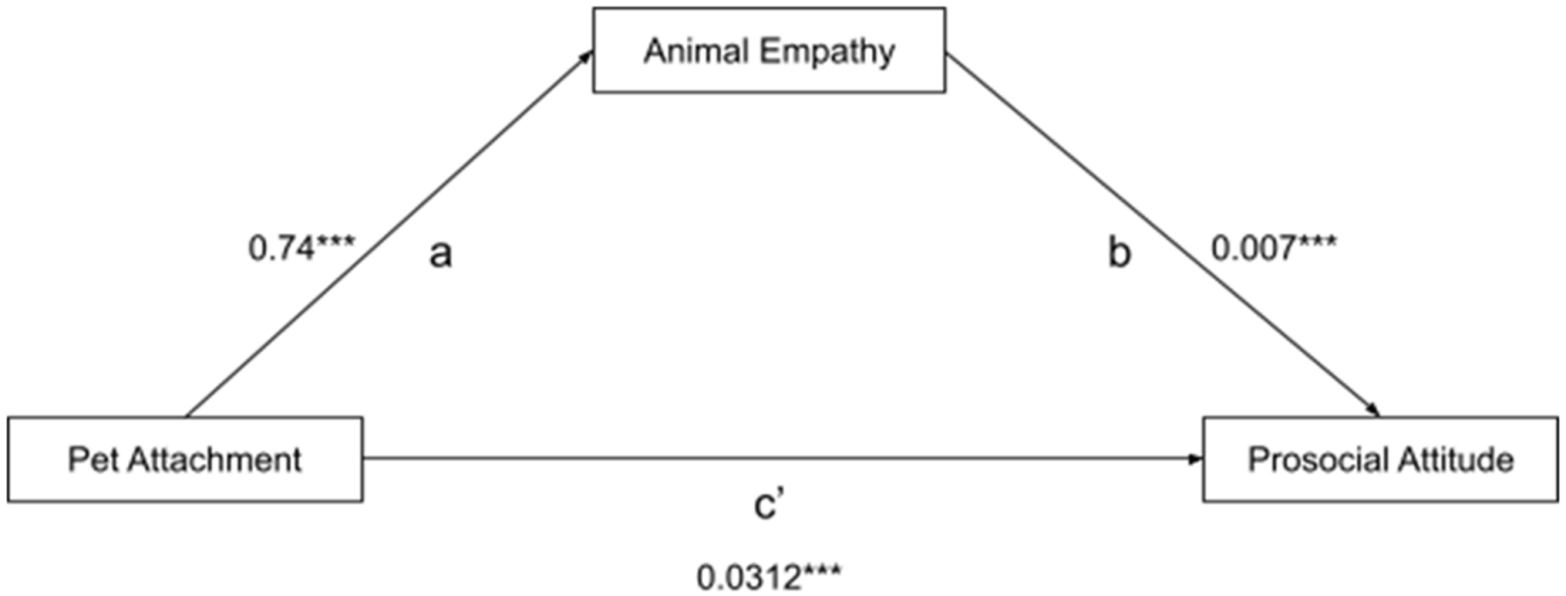
Mediation model of the relationship between pet attachment, animal empathy, and prosocial attitude.

Table 3 and Figure 2 show the influence of pet attachment on prosocial attitude as mediated by animal empathy. The study hypothesized that pet attachment would positively predict prosocial attitude, and further posited that empathy toward animals may mediate this relationship. Following Hayes bootstrapping analysis, the findings supported the hypothesis that pet attachment predicts animal empathy (*β* = 0.74, *p* < 0.001). Additionally, the results revealed that animal empathy predicts prosocial attitude (*β* = 0.007, *p* < 0.001). Overall, the indirect effect analysis indicated that animal empathy mediates the relationship between pet attachment and prosocial attitude (ab = 0.0058, *p* < 0.001), and it accounts 20.9% in the variance of prosocial attitude.

**Table 3:** Results of mediation analysis: total effect, indirect effect, *CF*, *t*-stat.

Path						
*a*	*b*	Total effect	Indirect effect	*CF*	*t*-statistics	Conclusion
0.74	0.007	0.0312	0.0058	95%	4.85	Animal empathy partially mediates the link between pet attachment and prosocial attitude.

Moreover, the total effect analysis demonstrated that pet attachment positively predicts prosocial attitude (*β* = 0.028, *p* < 0.001). Furthermore, the direct effect analysis revealed that even in the presence of animal empathy as a mediator, pet attachment still significantly impacts prosocial attitude (*β* = 0.0312, *p* < 0.001). These results suggest that animal empathy has a partial mediating influence on the association between pet attachment and prosocial attitude.

## Discussion

The study investigates the extension of the attachment-prosocial behavior relationship, mediated by empathy in human-human interactions, to human-animal interactions. The hypotheses posit: (1) a positive association between pet attachment and prosocial attitudes; (2) a positive link between pet attachment and empathy toward animals; (3) a positive correlation between empathy toward animals and prosocial attitudes; and (4) the mediation of the relationship between pet attachment and prosocial attitudes by empathy toward animals.

Our study confirms a positive association between pet attachment and prosocial attitudes, echoing findings from Hawkins et al. (2017) who also observed this link, albeit in children. Our research, involving adults, extends this notion, suggesting that attachment relationships can influence prosocial tendencies beyond childhood. Additionally, our results align with Costa Martins et al.’s (2022) systematic review, indicating that attachment relationships commonly contribute to the development of prosocial behaviors. Notably, our study expands this understanding into the domain of Human–Animal Interaction (HAI), suggesting that fostering prosocial attitudes can occur within HAI contexts, not limited to human-human interactions. Although our study demonstrates the predictive power of pet attachment on prosocial attitudes, the effect size appears relatively modest compared to previous findings by Hawkins et al. (2017).

This study also confirms the positive link between pet attachment and empathy toward animals, consistent with existing research (Rothgerber and Mican, 2014; Khalid and Naqvi, 2016; Hawkins et al., 2017; Jacobson and Chang, 2018; Gujarathi and Chatterjee, 2021) and attachment theory (Gillath et al., 2005; Mikulincer and Shaver, 2005; Shaver et al., 2016). Attachment theory posits that pet attachment fosters animal empathy through the Internal Working Model (IWM), shaped by interactions with caregivers (Zilcha-Mano et al., 2011; Shaver et al., 2016). This model consists of the self-perception (IWM of self) and perceptions of others (IWM of others) (Costa Martins et al., 2022; Sanctis and Mesurado, 2022; Xu et al., 2022), with the attachment figure representing entities beyond the self (Gillath et al., 2005; Zilcha-Mano et al., 2011). Observing attentive caregiving from pets influences individuals to perceive themselves as loved or valued, shaping the IWM of self, and recognizing others as deserving of care, shaping the IWM of others (Mikulincer and Shaver, 2005; Shaver et al., 2016). Consequently, attachment relationships with pets serve as a basis for cultivating empathetic concern toward animals (Gillath et al., 2005; Mikulincer and Shaver, 2005; Zilcha-Mano et al., 2011; Shaver et al., 2016).

Furthermore, the current study establishes a link between animal empathy and prosocial attitudes toward humans, revealing that empathy toward animals predicts prosocial attitude. While the impact of animal empathy on prosocial attitudes is significant, it remains relatively modest. Empathy is widely recognized as a catalyst for prosocial tendencies, a concept supported by previous research (Lockwood et al., 2014; Silke et al., 2018; Stern and Cassidy, 2018; Wice et al., 2020; Călin et al., 2021). Attachment theory suggests that individuals’ inclination toward prosocial behavior stems from the caregiving behavioral system (CBS), distinct from the attachment behavioral system (Gillath et al., 2005; Shaver et al., 2010). Unlike seeking proximity to attachment figures during distress, the CBS motivates individuals to provide support and serve as a secure base for distressed others. This altruistic nature of the CBS, activated by recognizing distress in others, extends beyond identity-driven motivations, accounting for care toward species beyond one’s own (Mikulincer and Shaver, 2005; Zilcha-Mano et al., 2011; Shaver et al., 2016).

The influence of animal empathy on prosocial attitudes toward humans may arise from individuals incorporating empathetic processes learned from animals into their interactions with humans, considering pets as representatives of others in attachment relationships (Shaver et al., 2016; Schoeps et al., 2020; Xu et al., 2022). Pets assume the role of attachment figures, shaping individuals’ mental representations or Internal Working Models (IWMs) of others, which may influence attitudes toward fellow humans (Young et al., 2018; Prato-Previde et al., 2022). Indeed, research indicates that empathetic processes toward animals and humans are intertwined, with animal empathy influencing empathetic responses toward humans in attachment relationships (Stoeckel et al., 2014; Vanutelli and Balconi, 2015; Gómez-Leal et al., 2021). Animal empathy may moderate the functions of the CBS, particularly in recognizing distress cues shared through mammalian nature, leading to the expression of prosocial attitudes (Gillath et al., 2005; Mikulincer and Shaver, 2005; Shaver et al., 2010, 2016).

Finally, the current study provides evidence supporting the hypothesis that animal empathy mediates the relationship between pet attachment and prosocial attitudes. Mediation analysis reveals that one pathway through which attachment to pets predicts prosocial behavior is by fostering animal empathy. Specifically, animal empathy has a significant yet modest partial mediating effect on the association between pet attachment and prosocial attitudes. These findings align with previous research by Thompson and Gullone (2008) and Knox et al. (2022), which also identified affective empathy as a mediator between pet attachment and prosociality, with partial mediation observed. In contrast, Schoeps et al. (2020) found that cognitive empathy fully mediated the relationship between attachment and prosocial tendencies in human-human relationships, contrasting with our focus on human–animal interactions.

The partial mediation of animal empathy suggests that other aspects of pet attachment may also mediate its link with prosocial behavior. Additionally, attachment theory posits that attachment relationships can influence prosocial attitudes independently of empathetic processes. The activation of the caregiving behavioral system (CBS), triggered by recognizing distress cues, may prompt prosocial tendencies even without heightened empathy. Research by Sundell (2019) suggests that individuals can recognize emotional cues and exhibit automatic matching responses to emotional stimuli, even without empathy. This implies that individuals may demonstrate prosocial behavior based on their innate ability to perceive and respond to emotional signals, triggering the CBS. However, the development of empathy may require learning and the absence of inhibiting factors.

## Conclusion

The study highlights the relationship between attachment and prosocial behavior, particularly in the context of human-animal interaction (HAI). Findings suggest that the emotional connection with pets provides a foundation for increasing prosocial attitudes toward humans. Pet attachment’s ability to predict prosocial behavior may be attributed to its impact on animal empathy. Individuals often regard their pets as attachment figures deserving of care, influencing them to empathize with their pets’ well-being during stressful circumstances. As animal empathy develops, a propensity toward prosocial behavior emerges, likely resulting from individuals incorporating learned empathetic processes from animals into their interactions with humans, viewing pets as representatives in their Internal Working Model (IWM) of others. The presence of animal empathy enables individuals to discern distress signals, particularly those common in mammalian nature, potentially activating the caregiving behavioral system (CBS). This activation of the CBS may prompt the expression of prosocial tendencies as individuals recall various caring behaviors.

The study results should be approached with caution due to several limitations. One limitation is the reliance on a specific animal empathy scale, which may have inadequate psychometric properties for Southeast Asia. Moreover, the scarcity of scales focusing on cognitive empathetic processes presents a notable constraint. Future research should aim to develop and validate an animal empathy scale tailored to the Southeast Asian context, incorporating cognitive processes. Another limitation is the sole reliance on pet attachment strength to assess prosocial attitude development, potentially overlooking other relevant aspects of human-animal interaction. Future studies should explore additional factors, such as attachment orientation within the pet bond, to gain a more comprehensive understanding of the development of prosocial attitudes in human-animal interactions (HAI). Additionally, research should focus on examining the relationship between empathy toward humans and prosocial attitudes toward animals. We also acknowledge that our sample may have biases, potentially limiting the generalizability to other populations of pet owners. Additionally, the reliance on self-reported data could introduce response bias. Future research should include more diverse samples to enhance applicability.

Despite these limitations, the study’s findings bear substantial theoretical and practical implications. Theoretically, the study extends attachment theory to encompass HAI, emphasizing the role of non-human relationships in understanding human social behavior. Furthermore, it underscores the mediating influence of animal empathy, suggesting that individuals demonstrating empathy toward animals are more likely to exhibit prosocial behaviors toward humans. This integration of empathy and prosociality implies a shared mechanism underlying prosocial behavior across diverse relationship contexts, emphasizing the complexity of human social behavior.

Practically, the study suggests that animal-assisted interventions (AAIs) hold promise for increasing prosocial attitudes, particularly in therapeutic settings where animals facilitate empathy and prosocial behavior, especially among vulnerable populations. Educational initiatives targeting empathy toward animals could also play a vital role in promoting prosocial attitudes and behaviors. By raising awareness about the significance of empathy toward animals and its broader impact on social interactions, schools, community organizations, and mental health institutions can contribute to promoting a more empathetic and prosocial society. Moreover, the study underscores the importance of cultivating positive relationships between humans and animals for the well-being of both parties. Animal welfare policies and practices should prioritize promoting healthy and empathetic interactions between individuals and their pets, recognizing the mutual benefits of such relationships for human and animal welfare.

## Data availability statement

The raw data supporting the conclusions of this article will be made available by the authors, without undue reservation.

## Ethics statement

The studies involving humans were approved by Central Luzon State University Ethics Review Committee (ERC Code: 2022-323). The studies were conducted in accordance with the local legislation and institutional requirements. The participants provided their written informed consent to participate in this study.

## Author contributions

JF: Conceptualization, Data curation, Formal analysis, Investigation, Methodology, Writing – original draft. ED: Conceptualization, Data curation, Formal analysis, Investigation, Methodology, Writing – original draft. LD: Conceptualization, Data curation, Formal analysis, Investigation, Methodology, Writing – original draft. LF: Conceptualization, Data curation, Formal analysis, Investigation, Methodology, Writing – original draft. YS: Conceptualization, Data curation, Formal analysis, Investigation, Methodology, Writing – original draft. EA: Conceptualization, Formal analysis, Investigation, Methodology, Supervision, Writing – review & editing.
